# Neonatal BCG vaccination is associated with enhanced T-helper 1 immune responses to heterologous infant vaccines

**DOI:** 10.1016/j.trivac.2013.11.004

**Published:** 2014

**Authors:** Daniel H. Libraty, Lei Zhang, Marcia Woda, Luz P. Acosta, AnaMae Obcena, Job D. Brion, Rosario Z. Capeding

**Affiliations:** a Division of Infectious Diseases and Immunology, University of Massachusetts Medical School, Worcester, MA, USA; b Department of Immunology, Research Institute for Tropical Medicine, Manila, Philippines; c Department of Medicine, Research Institute for Tropical Medicine, Manila, Philippines; d San Pablo City Health Office, San Pablo, Philippines; e Department of Microbiology, Research Institute for Tropical Medicine, Manila, Philippines

**Keywords:** BCG, Vaccines, T-cell, Th1, Neonate, Infant

## Abstract

Neonatal Bacille Calmette Guérin (BCG) vaccination has been reported to have beneficial effects beyond preventing infantile tuberculous meningitis and miliary disease. We hypothesized that BCG vaccine given at birth would enhance T-helper 1 (Th1) immune responses to the first vaccines given later in infancy. We conducted a nested case-control study of neonatal BCG vaccination and its heterologous Th1 immune effects in 2–3 months old infants. BCG vaccination at birth was associated with an increased frequency of interferon-γ (IFN-γ) producing spot-forming cells (SFC) to tetanus toxoid 2–3 months later. The frequency of IFN-γ producing SFC to polioviruses 1–3 also trended higher among infants who received BCG vaccination at birth. The frequency of IFN-γ+/tumor necrosis factor-α (TNF-α)+CD45RO+CD4+ T-cells upon stimulation with phorbol myristate acetate (PMA)/Ionomycin was higher in 2–3 months old infants who received BCG vaccination at birth compared to those who did not. The circulating frequency of forkhead box P3 (FoxP3)+ CD45RO+ regulatory CD4+ T-cells also trended lower in these infants. Neonatal BCG vaccination is associated with heterologous Th1 immune effects 2–3 months later.

## Introduction

The Bacille Calmette Guérin (BCG) vaccine is given to neonates in most countries to prevent infantile tuberculous meningitis and miliary disease. It is one of the most widely used vaccines worldwide. The efficacy of neonatal BCG administration has been linked to its ability to effectively induce a T-helper 1 (Th1)-polarized neonatal immune response [1]. Th1 cells are effector and memory CD4+ T-cells polarized to produce interferon-γ (IFN-γ). Neonates and infants generally have reduced Th1 responses to many intracellular pathogens and toxins [1]. Neonatal BCG vaccination has also been reported to reduce neonatal and infant mortality due to diseases other than tuberculosis [2-4]. Beneficial heterologous immune effects on antibody responses to some routine infant immunizations have been reported [5]. When given at age 2 months and at the same time as other routine infant vaccines, BCG has been reported to enhance the immune responses to some unrelated vaccines [6]. We hypothesized that BCG vaccine given at birth would enhance Th1 immune responses to the first vaccines given later in infancy. In order to examine this hypothesis, we conducted a nested case-control study of neonatal BCG vaccination. This study was within an ongoing clinical study of dengue virus infections during infancy in San Pablo, Laguna, Philippines. We found that BCG vaccination at birth was associated with some heterologous Th1 immune effects 2–3 months later, particularly an increased frequency of IFN-γ producing spot-forming cells (SFC) to tetanus toxoid.

## Materials and methods

### Ethics statement

The study protocol was approved by the institutional review boards of the Research Institute for Tropical Medicine, Philippines, and the University of Massachusetts Medical School. Mothers and their healthy infants were recruited and enrolled after providing written informed consent.

### Clinical study

The nested case-control study was drawn from an ongoing clinical study of dengue virus infections during infancy that has been previously described [7]. There were *n* = 13 infants who did not receive BCG vaccine between birth and 2 weeks of age; they received the BCG vaccine after their first infant immunization with the diphtheria/pertussis/tetanus toxoid vaccine (DPT) and oral poliovirus vaccine (OPV) (cases). These infants did not receive neonatal BCG because they were delivered at home in outlying areas. There were *n* = 38 age- and sex-matched infants who received BCG between birth and 2 weeks of age (controls). Vaccination dates were obtained from Expanded Programme in Immunization (EPI) cards. Clinical and epidemiological information were also collected at the study visit. Normalized child growth indicators were determined using World Health Organization (WHO) child growth standards. Peripheral blood mononuclear cells (PBMC) were collected from infants at the first study visit, at approximately 8–12 weeks old. PBMC were isolated using Histopaque^®^ density centrifugation and cryopreserved.

### IFN-γ ELISPOT

Briefly, cryopreserved PBMC (2–4 × 10^5^ cells per well) were thawed and seeded onto polyvinylidene difluoride membrane 96-well plates (Millipore) precoated with 5 μg/ml anti-IFN-γ monoclonal antibody (clone D1K; Mabtech). Stimuli were tetanus toxoid (EMD Calbiochem, 150 μg/ml), inactivated poliovirus vaccine (Sanofi Pasteur SA, 1/3.3 dilution), recombinant hepatitis B surface antigen (Genway Biotech, 27 μg/ml), phytohemagglutinin (PHA) (Sigma–Aldrich, 5 μg/ml), or media control (complete RPMI 1640/10% fetal calf serum). After 48 h of incubation (except PHA, 18–24 h incubation), cells were removed by washing with phosphate-buffered saline plus 0.05% Tween 20. Secondary biotinylated anti-IFN-γ monoclonal antibody (clone 7-B6-1; Mabtech) was added at 2 μg/ml and the plates were incubated for 2 h at room temperature. Plates were washed again and IFN-γ was detected with avidin–peroxidase (3420-2H, Mabtech) and substrate kit (NovaRed, Vector Laboratories). The frequency of IFN-γ-producing cells was determined by using the ImmunoSpot S4 Pro Analyzer and the ImmunoSpot Academic V.4 Software (Cellular Technologies Ltd.). Experiments were performed in triplicate wells.

### Flow cytometry

IFN-γ and TNF-α secreting, and FoxP3+, CD4+ T-cells in infant PBMC were identified by ICS (intracellular cytokine staining). PBMC were washed with media, and then left unstimulated or stimulated for 4 h with PMA/Ionomycin (BD Biosciences). The stimulations were done in the presence of 1 μl Brefeldin A (BD Biosciences). Cells were first stained with surface Ab to CD45RO (clone UCHL1), fixed and permeabilized with the FoxP3 buffer set (BD Biosciences), and then stained with Abs to CD3 (clone UCHT1), CD4 (clone SK3), CD8 (clone SK1), IFN-γ (clone B27), TNF-α (clone 6401.1111), and FoxP3 (clone 259D/C7) (all Abs from BD Biosciences). Cells were analyzed using a FACSAria flow cytometer (BD Biosciences). LIVE/DEAD^®^ Fixable Dead Cell Stain Kit (LDA) (Invitrogen) was used to exclude nonviable cells from analysis. Relevant cells were identified as LDA−/CD3+/CD4+/CD8−/CD45RO+ or CD45RO−/IFN-γ+ or TNF-α+ or FoxP3+ cells (Supplementary Fig. 1). Data was analyzed using FlowJo^®^ software (Treestar).

### Statistical analysis

The SPSS software package (version 20.0) was used for statistical analyses. Comparisons between two continuous variables were performed using the non-parametric Mann–Whitney *U* test. Comparisons between categorical variables were performed using the *λ*^2^ test. *P*-values <0.05 were considered significant. *P*-values ≥0.05 and <0.10 were considered a trend.

## Results

### Case-control study of neonatal BCG vaccination

We conducted a nested case-control study of the heterologous Th1 immune effects of neonatal BCG vaccination. The timing of vaccinations, age at the time of PBMC collection, and characteristics of infants in the case-control study are shown in Table 1. There were no significant differences in any measured variable between infants in the case and control groups.

### Neonatal BCG vaccination is associated with an increased frequency of IFN-γ producing cells to tetanus toxoid and polioviruses in 2–3 months old infants

**Fig. 1:**
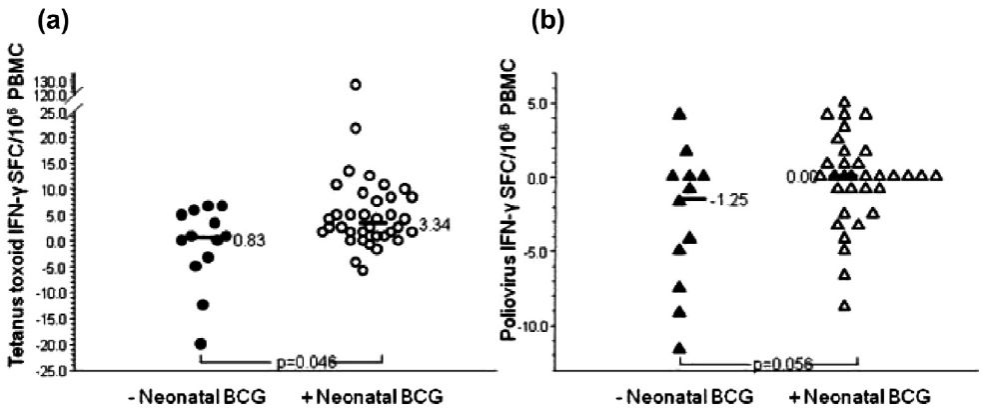
IFN-γ ELISPOT assays to tetanus toxoid and polioviruses 1–3. The frequencies of IFN-γ spot-forming cells (SFC)/10^6^ peripheral blood mononuclear cells (PBMC) from 2–3 months old infants to (a) tetanus toxoid and (b) inactivated poliovirus vaccine antigens are shown. Data points are the number of antigen-stimulated IFN-γ SFC – media control IFN-γ SFC. Negative values mean the number of antigen stimulated IFN-γ SFC was suppressed compared to media control. Bars are median values.

**Fig. 2:**
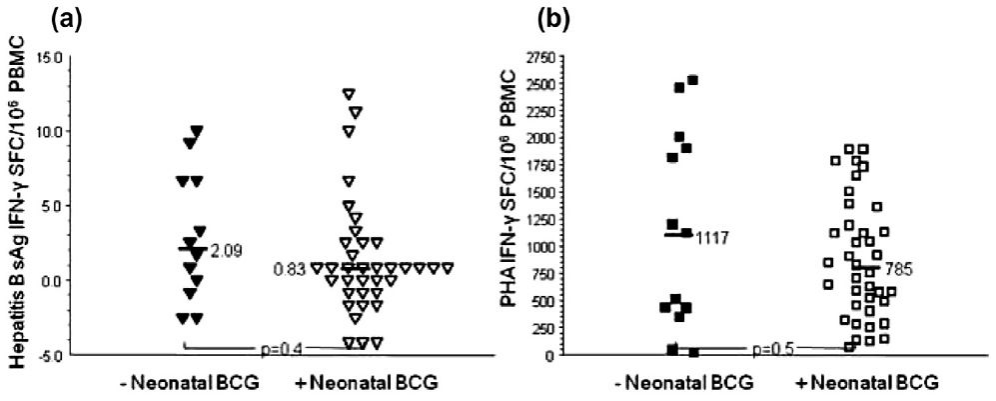
IFN-γ ELISPOT assays to hepatitis B surface antigen (sAg) and phytohemagglutinin (PHA). The frequencies of IFN-γ spot-forming cells (SFC)/10^6^ peripheral blood mononuclear cells (PBMC) from 2–3 months old infants to (a) hepatitis B sAg and (b) PHA are shown. Data points are the number of antigen-stimulated IFN-γ SFC – media control IFN-γ SFC. Negative values mean the number of antigen stimulated IFN-γ SFC was suppressed compared to media control. Bars are median values.

We performed IFN-γ ELISPOT assays to tetanus toxoid, polioviruses 1–3, hepatitis B surface antigen, and phytohemagglutinin (PHA) in the PBMC from 2–3 months old infants. Neonatal BCG vaccination was associated with an increased frequency of IFN-γ SFC to tetanus toxoid. The frequency of IFN-γ SFC to polioviruses 1–3 (inactivated poliovirus vaccine) also trended higher among infants who received BCG vaccination at birth (Fig. 1). There were no differences in the frequencies of IFN-γ SFC to hepatitis B surface antigen or PHA between the infants who received neonatal BCG vaccination and those who did not (Fig. 2). The frequencies of IFN-γ SFC to tetanus toxoid and polioviruses 1–3 were correlated (Spearman *r* = 0.61, *p* < 0.001). The frequencies of IFN-γ SFC to tetanus toxoid or polioviruses 1–3 did not correlate with IFN-γ SFC to either hepatitis B surface antigen or PHA.

### Neonatal BCG vaccination is associated with an increased frequency of IFN-γ+/TNF-α+ CD45RO+CD4+ T-cells upon PMA/Ionomycin stimulation in PBMC from 2–3 months old infants

**Fig. 3:**
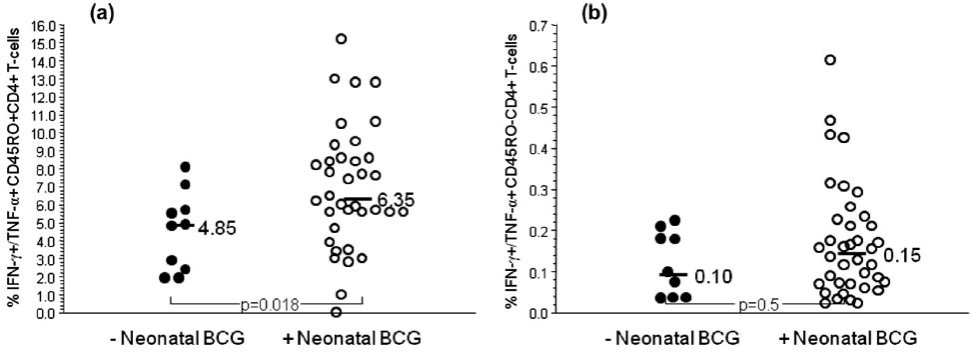
Intracellular cytokine staining for IFN-γ+/TNF-α+ CD4+ T-cells in the PBMC from 2–3 months old infants. The frequencies of phorbol myristate acetate (PMA)/Ionomycin stimulated – media control IFN-γ and TNF-α producing (a) CD45RO+CD4+ T-lymphocytes or (b) CD45RO−CD4+ T-lymphocytes are shown. Values are expressed as the % of either CD45RO+ or CD45RO− CD3+ CD4+ T-lymphocytes. Bars are median values.

The gating strategies for flow cytometry and intracellular cytokine staining (ICS) are shown in Supplementary Fig. 1. We examined IFN-γ and TNF-α production in CD4+ T-cells upon PMA/Ionomycin stimulation in the PBMC from 2–3 months old infants. BCG vaccination at birth was associated with an increased frequency of PMA/Ionomycin-stimulated IFN-γ+/TNF-α+ CD45RO+CD4+ T-cells during early infancy. There was no difference in the frequencies of PMA/Ionomycin-stimulated IFN-γ+/TNF-α+ CD45RO−CD4+ T-cells between infants who received BCG vaccination at birth and those who did not (Fig. 3). Essentially all infant CD4+ T-cells that produced IFN-γ upon PMA/Ionomycin stimulation also produced TNF-α.

### Neonatal BCG vaccination is associated with a decreased trend of circulating FoxP3+CD45RO+ regulatory CD4+ T-cells in 2–3 months old infants

**Fig. 4:**
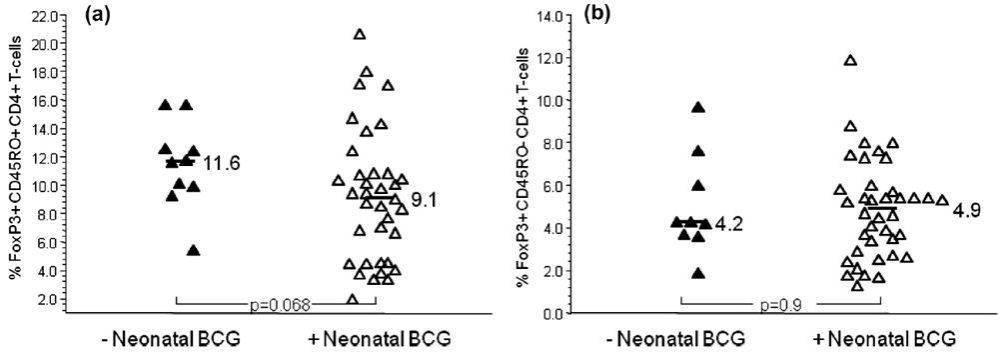
*Ex vivo* intracellular cytokine staining for forkhead box P3 (FoxP3)+ regulatory CD4+ T-cells in the PBMC from 2–3 months old infants. The circulating frequencies of FoxP3+ (a) CD45RO+ CD4+ T-lymphocytes or (b) CD45RO−CD4+ T-lymphocytes are shown. Values are expressed as the % of either CD45RO+ or CD45RO− CD3+ CD4+ T-lymphocytes. Bars are median values.

BCG vaccination at birth was associated with a decreased trend in the circulating frequency of FoxP3+CD45RO+CD4+ T-cells 2–3 months later. FoxP3+CD4+ T-cells are considered to be mainly regulatory T-cells (Tregs). There was no difference in the circulating frequencies of FoxP3+CD45RO− CD4+ T-cells between infants who received BCG vaccination at birth and those who did not (Fig. 4). The vast majority of all CD4+ T-cells, including FoxP3+ CD4+ T-cells, in the infants were CD45RO− (approximately 90%).

## Discussion

In a case-control study, we found that BCG vaccination at birth was associated with increased IFN-γ SFC to tetanus toxoid 2–3 months later. There was also an increased trend in the frequency of IFN-γ SFC to polioviruses. The frequency of IFN-γ+/TNF-α+ CD45RO+CD4+ T-cells upon stimulation with PMA/Ionomycin was higher in 2–3 months old infants who received BCG vaccination at birth compared to those who did not. The circulating frequency of FoxP3+CD45RO+ regulatory CD4+ T-cells also trended lower in these infants.

The tetanus toxoid and poliovirus vaccines induce predominantly Th1 cells during infancy [8-10], whereas neonatal hepatitis B vaccination is less reliant on Th1 induction [11,12]. Our IFN-γ ELISPOT results were consistent with these observations. The frequencies of IFN-γ SFC reflect a balance between Th1 and Th2 responses, which is why in some infants’ vaccine-stimulated responses were lower than media-stimulated responses. Neonatal BCG vaccination was associated with an increase in heterologous Th1 responses 2–3 months later to the infant tetanus toxoid and poliovirus vaccines. PHA induces IFN-γ production in both antigen-experienced (CD45RO+) and antigen-inexperienced (CD45RO−) CD4+ T-cells. The vast majority of infant CD4+ T-cells were antigen-inexperienced (CD45RO−), and there was no difference in the frequencies of IFN-γ+ CD45RO−CD4+ T-cells upon PMA/Ionomycin stimulation between infants who received BCG vaccination at birth and those who did not. As such, there was also no difference in the frequencies of PHA-stimulated IFN-γ SFC by ELISPOT.

In one report, BCG vaccination increased *in vitro* PBMC IFN-γ release to unrelated pathogens (*Staphylococcus aureus* and *Candida albicans*) for up to 3 months [13]. We postulate that BCG vaccination at birth primes neonatal dendritic cells to produce interleukin (IL)-12 p70 upon encountering intracellular pathogens or toxins. This Th1 polarizing effect of innate immunity lasts for several months. The ability of innate immune cells to acquire some memory and have cross-reactive immune effects has been termed “trained” immunity [14].

The observation that the circulating frequency of FoxP3+ CD45RO+ CD4+ T-cells trended lower among 2–3 months old infants who received BCG vaccination at birth is novel, but we are unable to distinguish whether this might be a cause or effect of Th1 polarization. One report demonstrated no significant difference in the *ex vivo* frequencies of FoxP3+CD4+ T-cells cells 4½ months after neonatal BCG vaccination [15]. Other limitations of this study are those of case-control studies in general. A prospective randomized study of neonatal BCG vaccination and its Th1 immune effects in 2–3 months old infants is planned. The fetal and early neonatal immune system is heavily Th2 biased [16,17]. This bias is felt to contribute to many suboptimal vaccine responses in neonates. The heterologous immune effects of BCG vaccination at birth provide important clues on how to improve neonatal and infant vaccine strategies.

## Supplementary Material

Figure S1

## Figures and Tables

**Table 1:** Characteristics of the case-control study population.

	Cases (−neonatal BCG)^a^ *n* = 13	Controls (+neonatal BCG)^a^ *n* = 38	*p*-value^b^
*Infant characteristics*			
Days between PBMC collection and BCG vaccination^a^,^c^,^d^	−14 (−83, −1)	60 (29, 90)	–
Days between PBMC collection and Hepatitis B vaccination #1^c^,^d^	48 (9, 78)	63 (50, 75)	*p* = 0.14
Days between PBMC collection and DPT vaccination #1^c^,^d^,^f^	14 (8, 35)	20 (13, 26)	*p* = 0.8
Days between PBMC collection and OPV vaccination #1^c^,^d^	14 (8, 35)	20 (12, 26)	*p* = 0.9
Age at time of PBMC collection (months)^c^,^d^,^e^	2.2 (2.0, 2.8)	2.6 (2.3, 2.8)	*p* = 0.3
Gender (male:female)	10:3	23:15	*p* = 0.3
Manner of delivery (vaginal delivery:C-section)	12:1	33:5	*p* = 1.0
Breastfed infants (%)^g^	69%	87%	*p* = 0.2
WHO weight-for-age *z* score^d^,^h^	−0.28 (−1.66, 1.09)	−0.50 (−2.01, 1.13)	*p* = 0.6
WHO length-for-age *z* score^d^,^h^	−0.38 (−1.89, 0.72)	−0.64 (−3.18, 0.62)	*p* = 0.5
WHO BMI-for-age *z* score^d^,^h^,^i^	0.00 (−1.37, 1.68)	−0.13 (−1.20, 2.10)	*p* = 1.0
WHO weight-for-length *z* score^d^,^h^	0.18 (−1.62, 1.91)	0.42 (−1.15, 2.83)	*p* = 0.6
*Mother characteristics*			
Maternal age at delivery (yrs)^d^	24.4 (21.4, 35.9)	21.8 (20.1, 26.1)	*p* = 0.11
Gravida^d^	2 (1, 3)	2 (1, 3)	*p* = 0.7
Paragravida^d^	2 (1, 3)	1 (1, 2)	*p* = 0.6
Highest level of education attained	Primary school-4	Primary school-8	*p* = 0.5
	High school-6	High school-25	
	College/University-3	College/University-5	
Number of persons in household^d^	5 (4, 6)	6 (5, 7)	*p* = 0.2

a BCG = Bacille Calmette Guérin vaccine.

b Comparisons between continuous variables were performed using the non-parametric Mann–Whitney *U* test; comparisons between categorical variables were performed using the *λ*^2^ test.

c PBMC = peripheral blood mononuclear cells.

d Values are median (95% confidence interval).

e DPT = diphtheria, pertussis, tetanus toxoid vaccine.

f OPV = oral poliovirus vaccine.

g Breastfed = exclusive or supplemental breastfeeding.

h WHO = World Health Organization.

i BMI = body mass index.
