# Supplementary figures and images for: Neonatal BCG vaccination is associated with enhanced T-helper 1 immune responses to heterologous infant vaccines
Source: Trials Vaccinol. Author manuscript; Available in PMC 2014 Mar 5. (PMC3943168; doi:10.1016/j.trivac.2013.11.004)

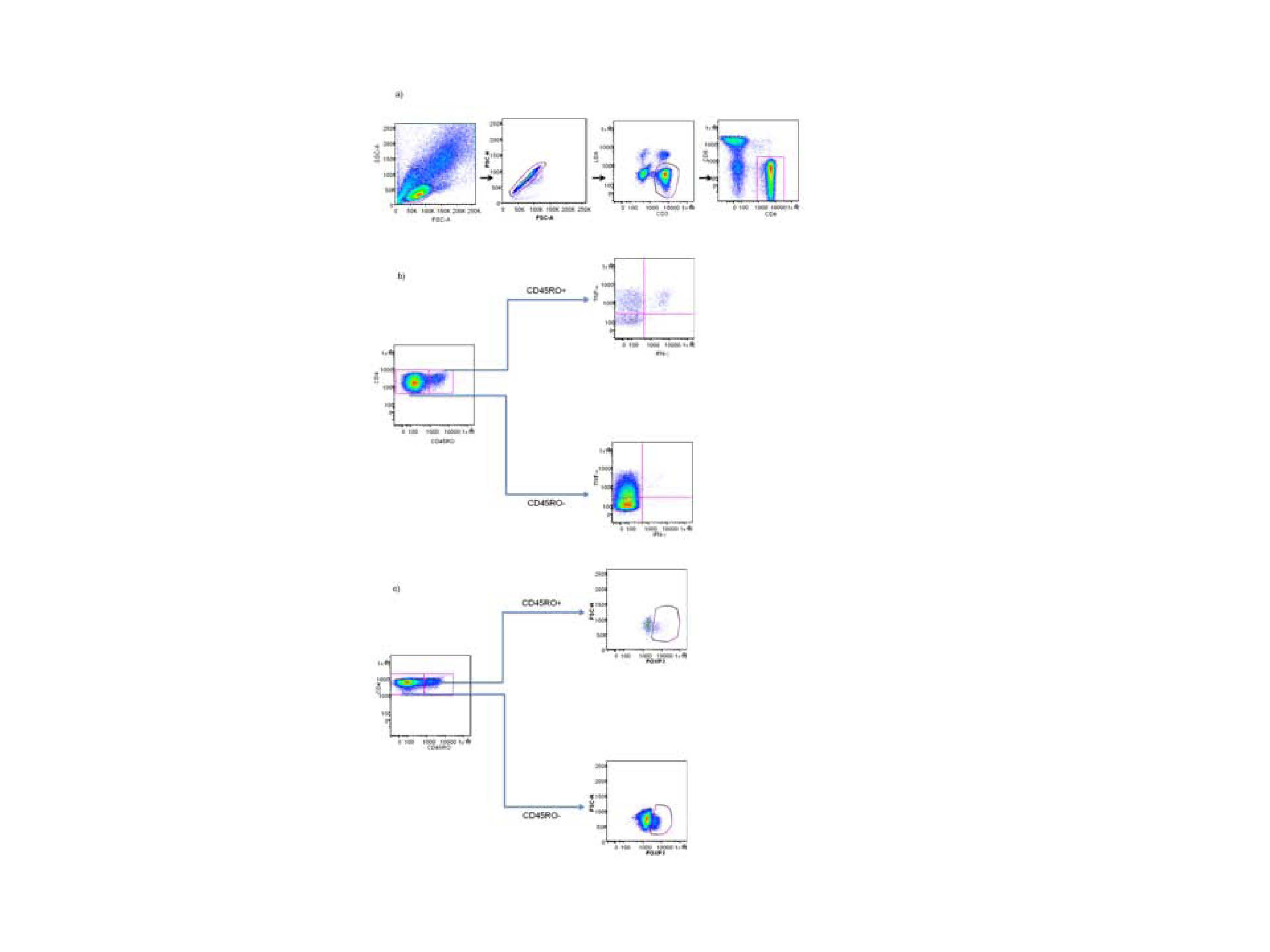

Supplement: Figure S1 [file NIHMS549692-supplement-Figure_S1.tif]
